# Repetitive Restricted Behaviors in Autism Spectrum Disorder: From Mechanism to Development of Therapeutics

**DOI:** 10.3389/fnins.2022.780407

**Published:** 2022-03-02

**Authors:** Junbin Tian, Xuping Gao, Li Yang

**Affiliations:** Peking University Sixth Hospital, Peking University Institute of Mental Health, National Clinical Research Center for Mental Disorders (Peking University Sixth Hospital), NHC Key Laboratory of Mental Health (Peking University), Beijing, China

**Keywords:** autism spectrum disorder, repetitive restricted behaviors, mechanism, therapeutics, neural circuit

## Abstract

Autism spectrum disorder (ASD) is a complex neurodevelopmental disorder characterized by deficits in social communication, social interaction, and repetitive restricted behaviors (RRBs). It is usually detected in early childhood. RRBs are behavioral patterns characterized by repetition, inflexibility, invariance, inappropriateness, and frequent lack of obvious function or specific purpose. To date, the classification of RRBs is contentious. Understanding the potential mechanisms of RRBs in children with ASD, such as neural connectivity disorders and abnormal immune functions, will contribute to finding new therapeutic targets. Although behavioral intervention remains the most effective and safe strategy for RRBs treatment, some promising drugs and new treatment options (e.g., supplementary and cell therapy) have shown positive effects on RRBs in recent studies. In this review, we summarize the latest advances of RRBs from mechanistic to therapeutic approaches and propose potential future directions in research on RRBs.

## Introduction

Autism spectrum disorder (ASD) is a common, heritable, and heterogeneous neurodevelopmental disorder characterized by deficits in social communication, social interaction, and repetitive restricted behaviors (RRBs). Kanner (1943) first described the autistic symptoms. The latest study has shown that the prevalence of ASD among American children aged 8 years was 1/44 or 2.27% (Maenner et al., 2021). RRBs are purposeless behavior patterns that interfere with normal behaviors and were confirmed as the core symptom of ASD in the Diagnostic and Statistical Manual of Mental Disorders, Fifth Edition (DSM-5) released by the American Psychiatric Association (2013).

Compared with social communication impairment, RRBs have gained less attention in ASD studies. RRBs occur in the early developmental stage and may interfere with the acquisition of essential life skills in the future. Furthermore, RRBs severely affect the quality of life and impose additional burdens on the family (Leekam et al., 2011; Wolff et al., 2014). Although behavioral intervention has achieved positive effects on RRBs (Boyd et al., 2012), the evidence of medication for RRBs remains insufficient. In this review, we summarize the latest studies on RRBs in ASD and suggest future directions in research on RRBs.

## Repetitive Restricted Behaviors

As an independent predictor of the prognosis of ASD (Troyb et al., 2016), the term “RRBs” is used to describe various behaviors and activities characterized by repetition, inflexibility, invariance, inappropriateness and frequent lack of obvious function and specific purpose, and highly restricted, fixated interests distinguished from the peers (Turner, 1999; Langen et al., 2011a). RRBs are thought pathological symptoms when they interfere with social relationships and impede daily activities. RRBs are non-specific symptoms observed in many other psychiatric disorders and developmental disabilities (Moss et al., 2009; Flores et al., 2011; Oakes et al., 2016; Evans, 2017). Moreover, RRBs also occur as common behaviors in typically developmental (TD) children, such as ritual behavior (Leekam et al., 2007; Arnott et al., 2010). In the section of ASD in DSM-5 (American Psychiatric Association, 2013), RRBs are divided into four subtypes: (a) Stereotyped or repetitive motor movements, use of objects, or speech. (b) Insistence on sameness, inflexible adherence to routines, or ritualized patterns of verbal or non-verbal behavior. (c) Highly restricted, fixated interests that are abnormal in intensity or focus. (d) Hyper- or hyporeactivity to sensory input or unusual interests in sensory aspects of the environment. Despite a lack of specific criteria to define different subtypes of RRBs, we can also identify the abnormal manifestation of repetitive behaviors depending on their characteristics and contexts in which they occur. For example, just turning lights and radios on or off is not considered RRBs, yet doing these repetitively without any specific purpose is recognized as abnormal RRBs. Because of the heterogeneity of RRBs, there are great challenges of deep understanding and completely assessing RRBs.

Firstly, there is rarely a consensus on the classification of RRBs by clinicians. In 1999, Turner classified RRBs into two types: “low-order” RRBs characterized by repetitive body movements (dyskinesia, convulsion, motor stereotypy, repeated manipulation of objects, and repetitive self-injury behavior) and “high-level” RRBs characterized by procedural and ritual behavioral patterns (insistence on sameness, resistance to change, repetitive language, and limited interest) (Turner, 1999). More studies divided RRBs into repetitive sensory motor (RSM) behaviors and insistence on Sameness (IS) behaviors (Cuccaro et al., 2003; Georgiades et al., 2010; Bishop et al., 2013). This two-factor model was consistent with the above classification described by Turner. However, the two-factor model has not been adopted in all studies. To date, many factor analysis studies have further examined the subtypes of RRBs by questionnaires designed for RRBs. The factor analysis based on repetitive behavior scale–revised (RBS-R) proposed a six-factor model: stereotyped behavior, self-injurious behavior, compulsive behavior, ritualistic behavior, sameness behavior, and restricted behavior (Bodfish et al., 2000; Esbensen et al., 2009). At present, based on the six-factor model of RBS-R, other researchers have also developed five-factor and three-factor models (Lam and Aman, 2007; Mirenda et al., 2010; He et al., 2019). The five-factor model merged the original subscales of ritualistic behavior and sameness behavior into one (ritualistic/sameness behavior subscale). This model seemed reasonable because both behaviors showed the same invariance and consistency needs and was more stable and reproducible than the original RBS-R (Lam and Aman, 2007). The three-factor model comprised compulsive ritualistic sameness behaviors, self-injurious behaviors, and restricted stereotyped behaviors. Mirenda et al. suggested that five- and six-factor models showed a better statistical fit than the three-factor model. However, the three-factor model also had advantages in genetic quantitative trait locus (QTL) analyses (Mirenda et al., 2010). He et al. (2019) considered the five-factor model preferable because the five-factor model had good psychometric characteristics and was more concise than the six-factor model. In addition, Leekam et al. (2007) obtained the four-factor model via repetitive behavior questionnaire-2 (RBQ-2). In a word, the classification criteria of RRBs are controversial. The variability of classification of RRBs may impact the consistency of results in different studies. That is to say, different measurement tools may divide a certain type of RRBs into different subcategories. For example, the item, arrange toys or other things in rows or patterns, was loaded into the subscale of preoccupation with restricted patterns of interest in RBQ-2, but the same-meaning item was allocated to the compulsive behavior subscale in RBS-R. This inconsistency may lead to the wrong conclusion regarding the more specific RRBs classification. Thus, it is necessary to compare various scales to confirm unified classification criteria and develop recognized assessment tools. These ensure results are comparable in different studies and further help to reveal more differences of RRBs in different populations, such as more severe self-injurious behavior in girls with ASD that cannot be found in studies using the two-factor model (Antezana et al., 2019).

Secondly, typically developing children also manifest some ritualistic, repetitive behaviors during early development (Evans et al., 1997; Leekam et al., 2007; Arnott et al., 2010). Then how can we differentiate RRBs between children with ASD and TD children? Usually, the RRBs in children with ASD are more excessive and diverse than those in TD children and result in severe impairments (Bodfish et al., 2000; Richler et al., 2007; Mandy et al., 2011; Harrop et al., 2014). Furthermore, following up repetitive behaviors across the developmental course is essential to determine whether it is aberrant. In TD children, repetitive behaviors are more common in toddlers than preschoolers (Kim and Lord, 2010). In early infancy, the stereotyped motor is considered a developmental manifestation of intrinsic central motor programs (Thelen, 1981). Repetitive behaviors may weaken with age in TD children (Larkin et al., 2017; Uljarevic et al., 2017; Sifre et al., 2021). However, the RRBs in children with ASD remain or aggravate with age (Leekam et al., 2007; Richler et al., 2010; Joseph et al., 2013).

## Assessment of Repetitive Restricted Behaviors

Early specific RRB symptoms predict the severity and outcome of ASD (Troyb et al., 2016; Miller et al., 2021). Moreover, two early studies indicated that preschool children with ASD displaying RRBs tended to have worse school-age language outcomes than those who did not exhibit RRBs (Charman et al., 2005; Paul et al., 2008). These findings emphasize the importance of early evaluation of all subtypes of RRBs.

There are three main methods to assess RRBs: parent interview, observation, and questionnaire. The autism diagnostic interview-revised (ADI-R), a semi-structured, standardized interview, is an acknowledged diagnostic tool of ASD (Lord et al., 1994). However, the items related to RRBs are scarce and concentrated in the dimension of restricted interest and behavior. Thus, some researchers contended that ADI-R was insufficient to cover all relevant RRBs occurring in children with ASD. The autism diagnostic observation schedule, 2nd edition (ADOS-2) combined with ADI-R, has been regarded as the gold standard for assessing children with ASD (Lord et al., 2012; McCrimmon and Rostad, 2014). Although some items about RRBs are included in the ADOS-2 algorithm, it is worth noting that this assessment may not find children’s RRBs in a limited time and single environment, thereby affecting the accuracy of assessment (Hus et al., 2014).

In addition, questionnaire is an excellent supplement to parent interview and observation. We summarize frequently used RRBs questionnaires and their relative strengths and weaknesses in Panel 1.

**PANEL 1:** Currently used RRB questionnaires.

• Repetitive Behavior Scale-Revised (RBS-R): This scale is the most frequently used to measure the severity of RRBs. The 43 items were compiled into six subscales: stereotyped behavior, self-injurious behavior, restricted behavior, compulsive behavior, ritualistic behavior, and sameness behavior (Bodfish et al., 2000). RBS-R has a good psychometric criterion. Some researchers have also developed five- and three-factor models (Lam and Aman, 2007; Mirenda et al., 2010; He et al., 2019), but their applicability needs to be proven in future studies. Considering comprehensive items and convenient use, RBS-R has a wide range of clinical applications (Lam and Aman, 2007; Mirenda et al., 2010; Bishop et al., 2013; He et al., 2019). • Aberrant Behavior Checklist (ABC): This is a caregiver rating scale used to assess behavioral problems in ASD (Aman et al., 1985; Kaat et al., 2014). Compared with other tools, ABC includes more comprehensive behavioral problems. Besides RRBs, it also investigates other aspects, such as emotional stability, attention, and hyperactivity. Currently, ABC applies to children and adults and is used for measuring the results of drugs and behavioral interventions in individuals with ASD (Owen et al., 2009; Bearss et al., 2013). Nevertheless, a potential disadvantage is that the stereotypic behavior subscale contains only seven items mainly describing stereotyped motor and limb movements. • Repetitive Behavior Questionnaire (RBQ): The RBQ is created for the sole purpose of assessing RRBs and includes 33 items (Honey et al., 2012). Twenty-nine items examine four subtypes of RRBs, including repetitive movements, sameness behaviors, repetitive use of language, and circumscribed interests. The four additional items consist of a summary item, which examines children’s overall interests or hobbies, and three open questions: the earliest repetitive activity, the most marked or noticeable behaviors, and the problematic repetitive behaviors. Based on RBQ, some researchers have developed RBQ-2 (Leekam et al., 2007) and RBQ-2A (Barrett et al., 2015) suitable for adults and children, respectively. RBQ checks the frequency of specific RRBs. Thus, it is very suitable to study the frequency or prevalence of RRBs. Moreover, three open questions also provide more information. So far, RBQ is not widely used in clinical practice.

## Neuropsychology of Repetitive Restricted Behaviors

### Cognition

In the early stages, executive function (EF) impairment was thought an explanation for RRBs, starting with Turner (1997, 1999). EF first develops in the early stages of development, approximately the end of the first year of life, and develops rapidly at the age of 2–5, which is in line with alterations of RRBs with age (Leekam et al., 2011). Numerous studies have supported a close connection between elevated RRB levels and EF impairments in children with ASD (Lopez et al., 2005) and TD children (Iversen and Lewis, 2021), such as set-shifting (Miller et al., 2015), inhibitory control (Mosconi et al., 2009), cognitive flexibility, and working memory (Van Eylen et al., 2015). An alternative view suggested that impaired EF was another manifestation of RRBs rather than an independent causative force driving RRBs. For example, the impairment of inhibitory control and set shifting seemed to be more related to the “high-order” RRBs, indicating that it might be that we were looking at the same general phenomenon (i.e., behavioral inflexibility or cognitive inflexibility) through different lenses (Mosconi et al., 2009; Van Eylen et al., 2015; Schmitt et al., 2018; Faja and Nelson Darling, 2019). Overall, RRBs can be indexed in many ways, including direct observations of behaviors, standardized rating scales, and neuropsychological tests of, for example, set-shifting or cognitive flexibility.

### Reinforcement and Habit

Organisms are motivated to seek reward stimuli (e.g., pleasant experiences or positive outcomes) or achieve specific goals, which increases the probability that specific behavior will be repeated (Wenzel and Cheer, 2018). This process is called reinforcement. Initially, ASD studies of reinforcement focused on social stimuli (Dawson et al., 1998; Klin et al., 2009). However, more attention has been given to the social motivation theory of autism in recent years (Chevallier et al., 2012). Children with ASD show diminished social motivation and a preference for non-social stimuli. This imbalance of motivations between non-social and social stimuli reflects the dysfunction of reward system (Dichter et al., 2012; Kohls et al., 2013), which may be the neurobiological basis of restricted interests (a subtype of RRBs) (Cascio et al., 2014; Clements et al., 2018; Kohls et al., 2018). Imaging studies revealed that the ventromedial prefrontal cortex (vmPFC) – ventral striatum (VS) – amygdala circuitry related to reward system seemed to be dysfunctional in ASD and underlay atypical reward responsiveness in individuals with ASD in part (Kohls et al., 2012; Langen et al., 2014). The activation of striatal regions increased in response to restricted interests in ASD (Clements et al., 2018). Similarly, Kohls et al. (2018) reported the stronger responsiveness of reward system to restricted interests rather than social rewards in children with ASD than TD children. Generally speaking, some types of RRBs may reflect, at least to a degree, reward-based processes (e.g., strong interest, motivation, and pleasure in response to unusual behaviors, objects, and activity) (Kohls et al., 2018). However, there is a lack of studies on the relationship between reward system and other subtypes of RRBs. Moreover, a part of individuals with ASD described that they felt pleasure when RRBs occurred, which urged them to do it again (Joyce et al., 2017). On the contrary, facing social communication, children with ASD had to confront the changing environments and unexpected events (Dawson et al., 1998). Thus, it is reasonable to presume that the preference for non-social stimuli reduces unpredictability and brings pleasant experiences to compensate for the anxiety and aversion of social communication in individuals with ASD. However, the contention is speculative and in need of empirical testing beyond a subjective sense of function (e.g., feeling pleasure).

In addition, reward-guided behaviors usually start as goal-directed actions that are controlled by the anticipation of the outcome. However, these behaviors can become stimulus-driven habits under certain conditions, which are not controlled by outcome expectancy (Yin and Knowlton, 2006). After achieving the same results via repetitive behaviors multiple times, we may focus less on the outcomes of actions, and goal-directed actions become automatized and habitual (Simmler and Ozawa, 2019). That is to say, goal-directed actions are controlled by their consequences, habits by antecedent stimuli (Yin and Knowlton, 2006). Alvares et al. (2016) found reduced goal-directed action control in individuals with ASD, which promoted habitual actions in an anxiety-inducing environment (e.g., social encounters). However, Geurts and De Wit (2014) did not find a disruption in the balance between goal-directed and habitual behavioral control in children with ASD. This inconsistency may be due to the age difference in the two studies (Alvares et al., 2016). Moreover, the corticostriatal connectivity is the neurobiological basis of the balance between habitual and goal-directed action control (Yin and Knowlton, 2006; Wit et al., 2012). Augustine et al. showed the reduced functional connectivity between the prefrontal and striatal regions (i.e., regions associated with goal-directed behaviors). However, the functional connectivity between motor/premotor cortex and striatal regions (i.e., regions critical for developing and regulating habitual behaviors) had no difference in children with motor stereotypies (a type of RRBs) compared to TD children (Augustine et al., 2021). A speculative contention was decreased prefrontal – striatal connectivity altered the balance between habitual and goal-directed action control, which resulted in enduring motor stereotypies. To sum up, it is unclear whether RRBs can be considered persistent and habitual actions based on a functional imbalance hypothesis referring to habitual and goal-directed action control. The notion is largely needed to be supported by empirical evidence.

### Habituation

Habituation is defined by an increasing reduction in behaviors and neural responses to repetitive stimuli, not caused by adaptation of sensory receptors or motor fatigue (Thompson and Spencer, 1966; Schmid et al., 2014). For example, repetitive affective and facial expression stimuli resulted in the habituation of automatic nervous systems and amygdala responses (Klorman et al., 1977; Klorman and Ryan, 1980; Breiter et al., 1996; Knight et al., 2005; Hare et al., 2008). In addition, habituation, in turn, facilitates children to pay more attention to the unknown from something acquainted, which promotes learning and adaptive responses to environmental changes (Groves and Thompson, 1970; Lloyd et al., 2014). Current studies supported the abnormal habituation in ASD (Guiraud et al., 2011; Swartz et al., 2013). Due to the abnormal habituation to normal input of sensory signals, individuals with ASD exhibited abnormal hyperresponsivity to environmental stimuli. Green et al. (2015) found that youth with ASD and sensory overresponsivity had attenuated neural habituation to stimuli in sensory cortices and the amygdala compared to the control and showed that this hyperresponsivity was due to failure to habituate. Hyperresponsivity to environmental stimuli was related to negative emotional reactions (e.g., anxiety) and highly uncertain perception of the environment (Uljarevic, 2013; Black et al., 2017; Vasa et al., 2018; Pickard et al., 2020). In addition, there is an apparent correlation between anxiety and RRBs. RRBs play a potential role in alleviating anxiety, and anxiety is an intrinsic motivator for repetitive behaviors (Joosten et al., 2009; Leekam et al., 2011; Rodgers et al., 2012; Spiker et al., 2012; Lidstone et al., 2014). Thus, we speculate RRBs may diminish the unpleasant emotional reactions due to the sensory hyperresponsivity and environmental uncertainty by some behaviors related to escape or avoidance in part. In conclusion, it is proposed that RRBs are coping strategies of hyperresponsivity to sensory stimuli caused by abnormal habituation. However, this contention is speculative and also lacks empirical support. It is essential to conduct more studies to explore the relationship between RRBs and habituation.

## Mechanism of Repetitive Restricted Behaviors

Autism spectrum disorder (ASD) is primarily caused by multiple genetic mutations that affect the structure and function of neural circuits. Various abnormities of brain regions and circuits are related to repetitive behaviors. In addition, the latest neurobiological and immunological findings suggest complex and diverse mechanisms of RRBs. Further understanding the mechanisms of RRBs helps to find more potential therapeutic targets.

### Cortico-Striatal-Thalamo-Cortical Circuit

Autism spectrum disorder (ASD) has been conceptualized as a brain network connectivity disorder (Just et al., 2004). The aberrant circuits predicted distinct RRBs in children with ASD (Supekar et al., 2021). Many studies focused on the role of the cortico-striatal-thalamo-cortical (CSTC) circuit in RRBs because this circuit is closely related to the execution of goal-oriented behavior. Interruptions or abnormalities (e.g., neuronal alterations and aberrant projections) in the CSTC circuit caused dysfunctional motor control (Lewis and Kim, 2009; Graybiel and Grafton, 2015). Previous and more comprehensive reviews have summarized neuroimaging (Wilkes and Lewis, 2018; Hiremath et al., 2021) and neurobiological studies (Gandhi and Lee, 2020; Vicente et al., 2020) of the role of CSTC in RRBs. In this section, we will review new findings of RRBs.

#### Neuroimaging of Cortico-Striatal-Thalamo-Cortical Circuit

Structural magnetic resonance imaging (MRI) studies found some abnormalities of the CSTC circuit with corresponding changes in RRBs. The orbitofrontal cortex (OFC) gray matter volume was positively associated with the severity of RRBs (Hegarty et al., 2020). However, right caudal anterior cingulate U-fiber volume was negatively associated with RRBs (Hau et al., 2019). Interestingly, sex differences in brain structure were associated with RRBs symptoms in autism. The female twin with more severe RRBs had increased thickness of the right intraparietal sulcus and decreased volume of the right orbital gyrus. However, increased volume of the bilateral pallidum was related to more severe RRBs in males (Van’t Westeinde et al., 2020). In addition, structural covariance describes the anatomical association in brain regions, which partially recapitulate networks of synchronized brain activity and is connected with the coordinated rates of developmental change in co-varying regions (Alexander-Bloch et al., 2013). Aberrant structural covariance in subcortical regions, such as thalami and basal ganglia, occurred in children with ASD and predicted the severity of RRBs, which suggested that abnormities of coordinating development of subcortical regions played an essential role in RRBs (Duan et al., 2020). Similarly, Mei et al. (2020) also reported that the structural covariation in brain areas associated with the CSTC circuit was significantly correlated with RRBs in individuals with ASD. A latest preclinical neuroimaging study on RRBs showed that reduced volume in key cortical and basal ganglia regions, including the motor cortex, striatum, globus pallidus, and subthalamic nucleus, was associated with repetitive behaviors in C58/J mice (Wilkes et al., 2020).

There were some new reports of functional connectivity changes of the CSTC circuit in recent years. For example, the over-connectivity pattern primarily in networks involving the fronto-temporal nodes related to RRBs occurred in individuals with ASD (Conti et al., 2017). Ma et al. (2021) showed the increased cortico-striatal intrinsic functional connectivities (iFC) with age in ASD and significant correlations between ADOS-RRB scores and iFC of the dorsal attention network-posterior cingulate cortex/precuneus. Furthermore, Akkermans et al. (2019) showed that increased functional connectivity between the left nucleus accumbens (NAcc) and a cluster in the right premotor cortex/middle frontal gyrus was correlated to more severe RRBs in children with ASD.

Langen et al. suggested the cortico-striatal circuit could be functionally divided into three “macro-circuits.” Each circuit was comprised of discrete, essentially non-overlapping subcortical structures, such as the striatum, globus pallidus, and thalamus, and received multiple inputs from functionally related and interconnected cortexes (Langen et al., 2011b). The CSTC circuit mainly included the sensorimotor circuit (comprising the motor and oculomotor loops), the associative circuit (dorsolateral prefrontal loop), and the limbic circuit (lateral orbitofrontal and anterior cingulate loops) (Groenewegen et al., 2003; Langen et al., 2011a). The abnormities of any circuit could give rise to different RRBs types. Some studies focused on these discrete loops. For example, Abbott et al. reported that the individuals with ASD and high RRBs showed depressed frontoparietal/limbic and motor/limbic circuit ratios. In other words, RRBs seemed to be linked to the imbalance of cortico-striatal connectivity, which showed increased connectivity of limbic circuits, but reduced connectivity of frontoparietal and motor circuits (Abbott et al., 2018). Moreover, complex motor stereotypies (CMS) were rhythmic, repetitive, fixed, and purposeless movements (Oakley et al., 2015). Augustine et al. (2021) found reduced functional connectivity between the prefrontal cortex and striatal regions in children with CMS. However, functional connectivity between motor/premotor cortex and striatal regions was no different from the control group. In a word, these findings offered evidence of the role of discrete loops in RRBs.

#### Neurobiology of Cortico-Striatal-Thalamo-Cortical Circuit

**FIGURE 1:**
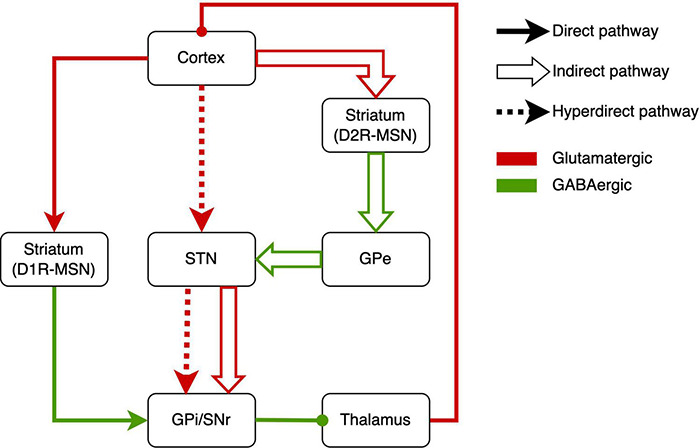
Schematic drawings of the direct pathway, indirect pathway, and hyperdirect pathway. GPe, external segment of the globus pallidus; GPi, internal segment of the globus pallidus; SNr, substantia nigra pars reticulata; STN, subthalamic nucleus; MSN, medium spiny neuron.

How this circuit regulates repetitive behaviors, here we describe the underlying neurobiological mechanisms of RRBs. Based on the neurobiological studies, the CSTC circuit is composed mainly of the direct pathway (cerebral cortex-striatum-internal segment of the globus pallidus/substantia nigra-thalamus-cerebral cortex) and indirect pathway (cerebral cortex-striatum-external segment of the globus pallidus-subthalamic nucleus-internal segment of the globus pallidus/substantia nigra-thalamus-cerebral cortex) (Figure 1) (Kim et al., 2016). There are two major classes of medium spiny neurons (MSNs) in the striatum [i.e., D1R-expressing direct pathway MSNs (dMSNs) and D2R-expressing indirect-pathway MSNs (iMSNs)], and MSNs respectively project to different brain areas (Figure 1). The behavioral result of activation of the direct pathway is motor activation/movements. However, activating the indirect pathway will reduce motor activity and movement (Calabresi et al., 2014). There was abundant evidence of dysfunction of direct and indirect pathways in ASD. Either of dysfunctions gave rise to the imbalance of both pathways (Kim et al., 2016; Gandhi and Lee, 2020), which might underlie RRBs (Lewis and Kim, 2009; Burguière et al., 2015; Monteiro and Feng, 2016).

Recently, some studies reported abnormal activation of the direct pathway in RRBs. In conditional knockout mice, the overactivation of dMSNs caused excessive self-grooming (a pathological repetitive behavior in mice), which suggested the role of direct-pathway deficiency in RRBs (Shonesy et al., 2018). Similarly, optogenetic activation of dMSNs also resulted in sustained and chronic repetitive behaviors (Bouchekioua et al., 2018). Engeln et al. (2021) found chemogenetic inhibition of dMSN can reduce repetitive rotations. In addition, the increased RRBs related to aberrant dMSNs have been found in Neuroligin 1 and Neuroligin-3 mutant mice (Rothwell et al., 2014; Espinosa et al., 2015).

Other studies revealed the indirect pathway role in RRBs. Shank3 deletion preferentially caused synaptic defects in iMSNs in Shank3B-KO mice, which provided direct evidence that a primary dysfunction of indirect pathway brought about the RRBs in ASD mice (Wang et al., 2017). In addition, Brandenburg et al. (2020) reported the increased dopamine type 2 gene expression in the dorsal striatum in postmortem brain tissue from an individual with ASD, which implied the alteration of indirect pathway in ASD. When it comes to the indirect pathway, STN, a vital part of the indirect pathway, has to be mentioned (Tanimura et al., 2011; Wilkes et al., 2020). In C58/J mice, reduced volume in STNs was associated with repetitive behaviors (Wilkes et al., 2020). The pharmacological studies on the indirect pathway also implied the indirect-pathway role in RRBs. For example, sub-chronic drug treatment targeting the indirect pathway reduced repetitive behavior in C58 mice and improved the STN dysfunction (Muehlmann et al., 2020). Similarly, adenosine A2A receptor agonist treatment attenuated increased grooming behaviors in BTBR mice (Amodeo et al., 2018). Selectively enhancing the indirect striatal pathway activation also corrected the RRBs in Shank3B-KO mice (Wang et al., 2017). Moreover, exposure to environmental enrichment retarded the development of stereotypy and recovered the decreased STN activation related to RRBs in high-stereotypy mice (Tanimura et al., 2010). Subsequently, the same team reported that this effect was based on the increased neuronal activation and dendritic spine densities in STN (Bechard et al., 2016). Besides, high-frequency stimulation at STN significantly alleviated RRBs in rodents (Aliane et al., 2012; Chang et al., 2016) and primates (Baup et al., 2008). However, there was a lack of neuroimaging studies that revealed the relation between RRBs and STN in humans. To sum up, the activations of direct and indirect pathways determine behavioral responses, and the imbalance of activations will give rise to RRBs.

The hyperdirect pathway, an integral component of the CSTC circuit, may be involved in the occurrence of RRBs (Figure 1). The STN is considered to receive fast monosynaptic projections from motor areas of the cortex via the hyperdirect pathway (Nambu et al., 2002). This pathway has been verified in humans (Brunenberg et al., 2012; Kelley et al., 2018) and animal models (Haynes and Haber, 2013; Averbeck et al., 2014). Activation of hyperdirect pathway will inhibit ongoing motor movements (Bahuguna et al., 2015). Increasing studies on the role of the hyperdirect pathway in inhibitory control have been reported in humans and animals (Eagle et al., 2008; Rae et al., 2015; Pasquereau and Turner, 2017; Jahfari et al., 2019). Cai et al. (2019) showed that the hyperdirect pathway predicted the inhibitory control in children. As mentioned earlier, inhibitory control may be another manifestation of RRBs. Thus, we suggested the hyperdirect pathway is related to RRBs, although there is a lack of direct evidence for the correlation between hyperdirect pathway and RRBs in ASD.

Moreover, the CSTC circuit is modulated by endogenous neuropeptides, including cannabinoids, opioids, and several other neurotransmitters. Shonesy et al. (2018) found that regulating endocannabinoid signaling in the direct pathway influenced the level of RRBs. Endogenous opioids in the frontal cortex (i.e., the starting site of projections in direct and indirect pathways) were negatively correlated to RRBs (Augustine et al., 2020). In addition, some studies have found abnormalities in the CSTC circuit in genetically mutated mice with particular signal transduction deficits, such as Dlg2 deletion mice (Yoo et al., 2020) and xCT −/− mice (Bentea et al., 2020). These studies gave support to the importance of the CSTC circuit in RRBs.

### Cerebellum

The cerebellum is related to sensorimotor processing and motor control (Bostan et al., 2010; Mosconi et al., 2015a). Increasing evidence suggests cerebellar connection dysfunction in ASD (Mosconi et al., 2015a,b), and these structural and functional alterations of the cerebellum are associated with RRBs (Rojas et al., 2006; Cheung et al., 2009; D’Mello et al., 2015; Wolff et al., 2017). The loss of Purkinje cells in the cerebellum may be the biological basis of RRBs (Al Sagheer et al., 2018). In this section, we summarize the new findings based on previous reviews (Wilkes and Lewis, 2018; Gandhi and Lee, 2020; Vicente et al., 2020).

Alteration of cerebellum structure related to RRBs has been reported in ASD. Srivastava et al. (2019) showed that cerebellar vermis volume reduced with high RRBs score and may predict severity of RRBs in Phelan-McDermid syndrome. However, a study of children at high risk of ASD found that high-risk infants have larger cerebellar at 4–6 months of age, and alterations in the volume are positively correlated with repetitive behaviors at 36 months. The study suggests that early cerebellar and subcortical volumes predicted repetitive behaviors in children (Pote et al., 2019). These inconsistent results may be due to the different ages of participants. Previous studies of younger children with ASD found larger total cerebellum (Sparks et al., 2002) and cerebellar white matter volume (Courchesne et al., 2001). In addition, RRBs were related to the volume of the crus II of the cerebellum in C58/J mice (Wilkes et al., 2020). All above data supported the cerebellum volume to be a potential biomarker for predicting RRBs severity.

Functional magnetic resonance imaging (fMRI) studies indicated aberrant connectivity between the cerebellum and cerebral cortex. Lidstone et al. collected resting-state fMRI from 105 children with ASD and found that elevated RRBs were associated with low right posterior cerebellum-left inferior parietal lobule (IPL) connectivity and high right posterior cerebellar-right IPL connectivity (Lidstone et al., 2021). Kelly demonstrated disrupted functional connectivity between the cerebellum and the medial prefrontal cortex (mPFC) in multiple mouse models of ASD-linked genetic mutations and individuals with ASD. Modulating the circuit from the right cerebellum crus1 area to the mPFC can lead to repetitive behaviors in Tsc1 mutant mice (Kelly et al., 2020).

Recently, the connection between the basal ganglia and cerebellum has increased attention. Two-way communication between the basal ganglia and cerebellum has also been proved in primates (Bostan et al., 2010). In addition, Chen et al. found a disynaptic pathway between striatum and cerebellum in mice. This short-latency pathway allowed rapid communication between the cerebellum and the basal ganglia. Thus, cerebellum can regulate the corticostriatal plasticity. Under pathological conditions, abnormal activity from the cerebellum was transmitted to the basal ganglia, which led to dysfunctional behaviors (Chen et al., 2014). Other studies also provided evidence of a powerful, short-latency pathway that connected the cerebellar dentate nucleus with the dorsolateral striatum (Bareš et al., 2015). The above reports were based on animal models. This connection also appears in humans. Milardi et al. found a direct route linking the dentate nucleus to the internal globus pallidus and the STN in healthy people (Milardi et al., 2016). Moreover, the basal ganglia and cerebellum were involved in different learning systems (i.e., reward-based learning and developing specific conditioned responses). Dasgupta et al. suggested their complementary roles in behavioral learning and the substantial bidirectional communication between these two brain structures. The combination of learning systems based on the basal ganglia and cerebellum allows for more stable and faster learning of goal-directed behavior than individual systems (Dasgupta et al., 2014). The imbalance between the two systems may lead to aberrant motor and non-motor functions (Subramanian et al., 2017). Based on these studies, the interaction between the cerebellum and basal ganglia may play an important role in RRBs. However, there is a lack of direct evidence to clarify the correlation between this connectivity and RRBs.

The cerebellar Purkinje cell (PC) dysfunction in the cerebellum may be the biological basis of RRBs (Mejias et al., 2019; Winkler et al., 2020). For example, the Shank3 mutant mouse exhibited significantly stereotyped behavior with fewer PC in cerebellar sub-regions (Matas et al., 2021). PC activation improved RRBs in PC-TSC1 mutant mice (Kelly et al., 2020). In addition, increased oxidative stress resulted in cerebellum dysfunction, which is associated with RRBs. Nadeem et al. (2019a,b) found that the deficiency of an adaptive antioxidant response in the cerebellum was related to increased repetitive behaviors in BTBR mice, and sulforaphane can restore this deficiency to improve the RRBs.

### Abnormal Immune Functions

Abnormal immune functions are related to ASD symptoms (Moradi et al., 2021). The immune dysfunction in ASD directly affects various neurodevelopmental and neurological processes, resulting in behavioral abnormality (Mead and Ashwood, 2015). In primate models of maternal immune activation (MIA), rhesus monkey offspring exposed to MIA *in utero* exhibited an increased frequency of motor stereotypies (Bauman et al., 2014; Rose et al., 2017). It has been demonstrated that the increased level of maternal autoantibody was associated with more severe RRBs. For example, exposure to endogenous maternal anti-Caspr2 antibody *in utero* led to robust RRBs in male mice (Bagnall-Moreau et al., 2020). Similarly, constant exposure to the autism-specific maternal autoantibodies throughout gestation result in apparent RRBs in C57BL/6J mice (Jones et al., 2020). In addition, offspring mice received a single intraventricular injection of IgG from two mothers of children with ASD on embryonic day 14 displayed RRBs (Camacho et al., 2014). Martin et al. (2008) found treatment with IgG from mothers of children with ASD induced offspring to exhibit whole-body stereotypies in rhesus monkeys. However, Bauman et al. (2013) did not find any alterations of repetitive behaviors in rhesus monkeys injected with human IgG isolated from mothers of children with ASD six times during early and mid-gestation. The inconsistency of offspring behaviors was probably due to the route and number of injections administered, different animal models (Watson and Platt, 2012), the pregnancy time of the injection, the alteration of testing circumstances (Hutt and Hutt, 1965; Runco et al., 1986), and most importantly, the source of IgG used (Salloum-Asfar et al., 2019). Further studies are needed to determine the influence of these factors. However, there was no doubt that maternal antibodies played a role in RRBs. In addition, BTBR mice exhibited increased oxidative stress and insufficient enzymatic antioxidant responses associated with autistic repetitive behaviors (Nadeem et al., 2019a). In recent years, more clinical studies have found that higher levels of proinflammatory factors, such as IL-1β (Ashwood et al., 2011b) and IL-10 (Meyer et al., 2008), were related to more severe RRBs (Ashwood et al., 2011a; Careaga et al., 2017; Rose et al., 2017). Regulating immune pathways in BTBR mice reduced RRBs with decreased levels of proinflammatory cytokines (Zhang et al., 2019). In summary, proinflammatory factors may be the mediator between immune dysfunction and RRBs. Many pharmacological studies of RRBs were based on immune abnormalities in ASD (Ahmad et al., 2019). For example, Mirza and Sharma (2019b) found that pioglitazone reduced oxidative stress and nerve inflammation in related brain regions and improved propionic acid-induced neurobehavioral and biochemical impairments in rats. Li et al. (2020) found that aberrant eating behaviors and high food-specific IgG antibody concentrations were related to more severe RRBs in children with ASD. In addition, the ketogenic diet could improve the high levels of repetitive behavior in male C57Bl/6 mice affected by MIA (Ruskin et al., 2017). At present, most studies were based on animal models with ASD, and a few measured the indicators of proinflammatory factors (Bryn et al., 2017). Moreover, more scholars focused on the relationship between immune dysfunction and abnormal gut microflora in individuals with ASD (Moradi et al., 2021). Based on the above findings, related immune pathways may become one of the therapeutic targets of RRBs.

### Other Potential Neural Mechanism

Most studies of the mechanism of RRBs were conducted in animal models with related genetic mutations. Researchers observed the typical core symptoms of ASD in various mutated mice (Satterstrom et al., 2020). Neurotransmitters, such as glutamate and γ-aminobutyric acid (GABA), regulated the balance of excitation and inhibition (E/I) in the brain (Cai et al., 2017). The increased excitatory signals and decreased inhibitory interneurons would induce RRBs in ASD animals (Rinaldi et al., 2007; Gogolla et al., 2009). Moreover, modulating the level of transmitters could change RRBs (Rhine et al., 2019). Abnormal neurotransmitter systems of brain areas related to RRBs would give rise to RRBs (Peca et al., 2011; Bentea et al., 2020), such as glutamate receptor-interacting proteins 1/2 (Grip1/2) (Mejias et al., 2019), metabotropic glutamate receptor 5 (mGluR5) (Silverman et al., 2010; Luo et al., 2018), and GABA_A_ receptor (Yoshimura et al., 2017). Yang et al. (2021) reported that acute administration of GABA-A or/and GABA-B receptor agonists could palliate repetitive behaviors in ASD mice.

Serotonin (5-hydroxytryptamine, 5-HT) plays a complex role in regulating neural circuits during prenatal and postnatal development (Whitaker-Azmitia, 2001; Veenstra-VanderWeele et al., 2012; Wirth et al., 2017). The alteration of the 5-HT neurotransmitter system in the brain has been reported in animal models and individuals with ASD (Muller et al., 2016). The studies of treatment with selective serotonin reuptake inhibitors (SSRIs) produced inconsistent results on RRBs (Costa et al., 2018; Reddihough et al., 2019; Herscu et al., 2020). Diverse 5-HT receptors have different effects on RRBs. For example, the blockades of 5-HT_2A_ receptor (Amodeo et al., 2017) and 5-HT_6_ receptor (Amodeo et al., 2021) reduced RRBs. Decreased activation of 5-HT_1A_ also achieved the same effect (Chugani et al., 2016). However, activating the 5-HT_7_ receptor reversed repetitive behaviors in Fragile X syndrome (Costa et al., 2018). The current studies on serotonin were based on animal models, and more complete studies in humans will confirm the relationship between 5-HT and RRBs.

Increasing studies related to RRBs focused on other neural signalings, such as dopaminergic signaling (Lee et al., 2018b; Venkatachalam et al., 2021), cannabinoid signaling (Marco et al., 2011; Fyke et al., 2021; Nezgovorova et al., 2021), mammalian target of rapamycin (mTOR) signaling (Burket et al., 2014; Chugani et al., 2016; Wu et al., 2017), adenosine signaling (Ansari et al., 2017; Lewis et al., 2019), and histamine signaling (Eissa et al., 2018; Eissa et al., 2020b; Venkatachalam et al., 2021). These signal molecules may play a role in regulating synaptic transmission in brain regions related to RRBs, and multiple signaling pathways might be involved in the pathological process simultaneously (Eissa et al., 2019; Eissa et al., 2020a; Muehlmann et al., 2020; Eissa et al., 2021; Venkatachalam et al., 2021).

## Treatment and Intervention of Repetitive Restricted Behaviors

In recent years, the number of studies on RRB treatment and intervention has increased. There is no recognized drug intervention for RRBs at present, and behavioral intervention remains the most effective and safe strategy for RRBs treatment. This section reviews the recent advance in drug intervention, supplementary therapy, and other potential therapies.

### Drug Treatment

Although no evidence-based effective medicine for RRBs in ASD has been proposed, some drugs based on new psychopharmacological mechanisms or molecular targets have shown potential benefits in early studies. Given the substantial individual differences in clinical response and side effects observed in current studies, more studies are needed to verify these findings.

#### Antipsychotic Drug

Risperidone and aripiprazole, the atypical antipsychotic drugs acting on the D2 dopamine receptor, have been approved to reduce irritability, agitation, aggression, and self-harm in ASD by the Food and Drug Administration (FDA) (McCracken et al., 2002; Owen et al., 2009). Although risperidone has been reported to reduce RRBs in salt-induced kinase 1 (SIK1)-mutant mice via attenuating neural excitability and excitatory synaptic transmission (Badawi et al., 2021), a meta-analysis published in 2020 showed that antipsychotics were not beneficial to RRBs in clinical trials (Yu et al., 2020). Subsequently, another meta-analysis including more RCTs pointed out a slight improvement in RRBs after administration of antipsychotics (Zhou et al., 2021). This finding has no practical value because clinicians must weigh these moderate benefits of antipsychotics against the considerable side effects (Correll et al., 2006). Compared with other clinical pharmacological trials of ASD, the studies of antipsychotics have a smaller sample size and more significant heterogeneity in estimated treatment effect.

#### Oxytocin

A great number of studies reported the critical role of oxytocin in human social interaction (Insel et al., 1999; Yatawara et al., 2016). Intranasal administration of the neuropeptide oxytocin (IN-OT) has been regarded as a potential therapy for the core symptoms of ASD. However, its effect in RRB has received less attention. An early study revealed oxytocin infusion reduced RRBs in adults with autism and Asperger syndrome (Hollander et al., 2003). This invasive method is not ideal and replaced by IN-OT for researchers and clinicians (Guastella et al., 2013). After 4 weeks of daily oxytocin administration (24 IU/day), RRBs were significantly reduced in 40 adult men with high-functioning autism (Bernaerts et al., 2020). Interestingly, a preliminary trial showed that 6 weeks of IN-OT had a significant effect on social communication rather than RRBs in 18 men with ASD (Watanabe et al., 2015). However, the same research team found that oxytocin reduced ADOS-RRB score in a larger sample (*n* = 106), which applied the same study design (Yamasue et al., 2020). This difference may be caused by sampling error and less placebo effect on RRBs related to less expectation for effects. In the same year, another randomized controlled trial (RCT) study also verified the benefits of oxytocin to RRBs (Alaerts et al., 2020). Regarding the underlying neural mechanism of the IN-OT effect, Alaerts et al. (2020) suggested that IN-OT might cause long-term alterations in the internal functional connectivity of the amygdala to the OFC, which was related to RRBs improvement. In addition, Watanabe et al. (2015) reported that the improvement of the core social symptoms in ASD was accompanied by oxytocin-induced enhancement of task-independent resting-state functional connectivity between the anterior cingulate cortex (ACC) and dorsomedial prefrontal cortex. As an increasing number of trials have evaluated the clinical response of multiple doses of IN-OT in ASD, Peled-Avron et al. (2020) conducted a meta-analysis that showed that IN-OT was well tolerated and supported that oxytocin could improve RRBs in ASD, although the effect size was small. However, some studies with small sample sizes have not found the benefits of oxytocin to RRBs (Anagnostou et al., 2012; Dadds et al., 2014; Guastella et al., 2015; Kosaka et al., 2016), it is necessary to conduct multi-center RCT studies with a larger sample and focus on the improvement of RRBs.

#### Bumetanide

Bumetanide is an effective diuretic. As mentioned above, GABAergic signals play a vital role in regulating RRBs (Cellot and Cherubini, 2014). Convincing evidence has shown that defects in inhibitory GABAergic signals led to ASD, and the level of GABAergic inhibition depended on the concentration of intracellular chloride [(Cl-)i] (Schulte et al., 2018). NKCC1 (Na-K-Cl cotransporter 1) is the primary transporter responsible for regulating (Cl-)i, and its activity controls the level of chloride in neurons, which further affects the post-synaptic effect of GABAergic transmission (Schulte et al., 2018). Bumetanide, a selective NKCC1antagonist (Ben-Ari, 2017; Kharod et al., 2019), could restore GABAergic inhibition and weaken behavioral and electrophysiological characteristics in various diseases (e.g., ASD and Fragile X syndrome) by regulating the concentration of neuronal chloride (Payne et al., 2003; He et al., 2014; Kaila et al., 2014; Tyzio et al., 2014; Juarez-Martinez et al., 2021). However, there is a lack of reports on the effects of bumetanide on RRBs in animal models. Increasing studies exhibited a positive effect of bumetanide in children with ASD or Fragile X syndrome (Lemonnier et al., 2013; Du et al., 2015; Zhang et al., 2020; Dai et al., 2021). After a pilot study reported the benefits of bumetanide to RRBs (Lemonnier and Ben-Ari, 2010), Lemonnier et al. conducted two RCT studies to test bumetanide in 60 and 88 patients. Both trials showed a significant reduction in scores of RRBs (Lemonnier et al., 2012; Lemonnier et al., 2017). Similarly, another Phase-2 Superiority Trial also revealed significant effects on RRBs in children aged 7–15, despite no superior effects on the primary outcome of social communication and social interaction (Sprengers et al., 2021). Crutel et al. (2021) described a design of two Phase III studies to evaluate the efficacy/safety of bumetanide oral liquid in ASD, which will provide strong evidence to support the benefits of bumetanide to RRBs. In addition, bumetanide could improve emotional face perception and increase the time spent in spontaneous eye gaze in ASD, with alterations of the activation level in corresponding brain regions (Hadjikhani et al., 2015; Hadjikhani et al., 2018). Current studies were mainly conducted in children and adolescents under 18, and this potential effect in adults should be further verified.

#### Other Drugs

Based on the possible neurobiological mechanism of RRBs, some emerging treatment methods, such as pioglitazone (Capano et al., 2018), pioglitazone (Chugani et al., 2016), intranasal administration of vasopressin (Parker et al., 2019), and IGF-1 injection (Kolevzon et al., 2014), could significantly improve RRBs in individuals with ASD. Some anti-inflammatory drugs targeting abnormal immune functions in ASD showed the benefits to RRBs, such as memantine plus risperidone (Ghaleiha et al., 2013), org 2766 (a synthetic analog of the adrenocorticotrophic hormone) (Buitelaar et al., 1990; Buitelaar et al., 1992), and celecoxib plus risperidone (Asadabadi et al., 2013). In 2020, a meta-analysis of pharmacological interventions for RRBs in ASD included 64 different trials conducted before November 2019. Except for the drugs mentioned above, divalproex sodium, leucovorin, and guanfacine as monotherapies have a more significant positive effect on RRBs of ASD (Zhou et al., 2021). Most findings of the above drugs were based on studies with a small sample and required to clarify their effects further. No pharmacological drug has shown significant clinical benefits and a solid evidence base of effectiveness.

#### Preclinical Pharmacological Studies

More drug studies are in preclinical stage, and a variety of ASD animal models have become the essential tools for preclinical studies (Lewis et al., 2007), which provides the theoretical basis for following clinical trials. Increasing drug trials in animal models are based on the hypotheses of potential mechanisms in ASD, especially abnormal neurotransmitter/neuromodulator systems. In *SHANK3* mutant mice, acute administration of tandospirone, a 5-HT_1A_ receptor agonist, reduced self-grooming behavior (Dunn et al., 2020). In addition, N-methyl-D-aspartate (NMDA) receptor plays an important role in the balance of E/I and postnatal low-dose MK-801, an NMDA receptor blocker, improved ASD-related behaviors in valproic acid (VPA)-treated rats (Kim et al., 2017; Mohammadi et al., 2020). Another potential function mechanism of the NMDA receptor antagonist was to ameliorate immune dysfunction. For example, dextromethorphan rescued the impaired behavioral patterns in VPA-induced autistic rats and decreased the levels of various oxidative stress and inflammatory markers (Singla et al., 2021).

Another area that receives much attention is the drugs targeting abnormal immune function. 5-aminoisoquinolinone (5-AIQ) has the effects of neuroprotection and down-regulated inflammatory responses (Alhosaini et al., 2021). In BTBR mice, the 5-AIQ treatment significantly prevented self-grooming and marble burying behaviors and ameliorated neuroimmune dysfunctions (Ahmad et al., 2020). Similarly, the benefits to RRBs were reported in the study of the administration of catechin hydrate and pioglitazone in VPA-induced rats (Mirza and Sharma, 2019a; Mehta et al., 2021) and sulforaphane in BTBR mice (Nadeem et al., 2019b). They corrected immune dysfunction and oxidant-antioxidant imbalance in periphery and brain in mice. Beyond that, Zhang et al. (2019) showed that folic acid reduced RRBs in BTBR mice via mitigation of oxidative stress, inflammation, and ferroptosis. Another nutritional supplement of gestational B-vitamin alleviated mitochondrial damage in the hippocampus and PM2.5-induced autism-like behaviors in mice offspring (Wang et al., 2019).

Since a large proportion of people with neurodevelopmental disorders such as ASD are disturbed in their daily sleep/wake cycles (Robinson-Shelton and Malow, 2016). In response to this phenomenon, researchers tried melatonin treatment in CNTNAP2 KO mice and found that it improved excessive grooming in mice (Wang et al., 2020). Furthermore, MTHFR polymorphism was associated with an increased risk of ASD, and the offspring of Mthfr + / − mice (Pu et al., 2013), whether wild-type or heterozygous, exhibited autism-like behaviors. It is surprising that after 14 days of choline supplementation, the characteristics of RRBs were offset (Agam et al., 2020). In addition, other supplementary treatments showed promising effects in ASD animals. For example, abnormally high levels of homocysteine (Hcy) were considered to have a relation with ASD (Kałużna-Czaplińska et al., 2013). Administration of betaine, a methyl group donor in Hcy metabolism, significantly ameliorates RRBs in VPA-induced autistic mice (Huang et al., 2019). Furthermore, exposure to VPA might alter zinc metabolism resulting in a transient deficiency of zinc. Cezar et al. (2018) showed that zinc supplements reduced the transient zinc deficiency and prevented VPA-induced RRBs in rats. Autistic children with similar genetic or metabolic alterations would benefit from similar supplementary treatment if these results are replicated. Other drug studies showed initial outcomes of RRBs in animal models (Bhandari and Kuhad, 2015; Luhach et al., 2021). For example, treatment with medical cannabis alleviated RRBs by over 70% in Shank3 mice (Poleg et al., 2021). Administration of beta-carotene (Avraham et al., 2019; Avraham et al., 2021) and curcumin (Zhong et al., 2020) reduced RRBs in BTBR mice. However, the limitations are that these drugs have multi-target effects and their specific mechanisms are unclear, hindering their use in clinical trials. Moreover, some drugs approved to treat other diseases revealed a new therapeutic effect on RRBs (Román et al., 2021; Ryu et al., 2021; Wu et al., 2021). Chinese herbal medicine also positively influenced RRBs in BTBR mice (Park et al., 2021).

### Behavioral Intervention

Behavioral intervention is still the most effective and safest intervention for RRBs. The behavioral intervention for RRBs has been comprehensively reviewed elsewhere (Odom et al., 2010; Boyd et al., 2012; Harrop, 2015; Kodak and Bergmann, 2020). This section only reviews the latest reports about the comprehensive treatment model (CTM) (Odom et al., 2010).

At present, the most widely used CTMs include the Denver Model, Structured Teaching (TEACCH), and Early Intensive Behavior Intervention (EIBI). Behavioral parent training (BPT) is considered the first choice of treatment for young children with disruptive behaviors (Kaminski and Claussen, 2017), and parent-child interaction therapy (PCIT) is one of the most supported evidence-based BPTs. After implementing PCIT, 16 individuals showed a significant improvement in RRBs compared to the control group (Parladé et al., 2020). Besides, self-management intervention and pivotal response treatment (PRT) were implemented in three young children with ASD, and results showed improvements in children’s higher-order RRBs and interactions with parents (Lin and Koegel, 2018). However, one limitation should also be considered: CTM is a multitarget intervention, and most studies assessed RRBs as one of the secondary results; therefore, limited high-quality research has reported on its efficacy on RRBs.

### Supplementary Therapy

Abnormal eating habits in children with ASD play a potential role in exacerbating ASD symptoms (Peretti et al., 2019). In recent years, increasing studies focused on the potential value of nutritional supplements in ASD, but limited evidence supported their effectiveness on RRBs.

#### Vitamin D

Studies have reported decreased vitamin D levels in the blood of patients with ASD (Wang T. et al., 2016). Vitamin D3 seemed to have therapeutic potential in ASD (Jia et al., 2015). After 3-month vitamin D3 supplementation, RRBs improved in 37 children with ASD, particularly in younger children (Feng et al., 2017). Another study involving 83 children with ASD also found an improvement in RRBs after 3-month treatment (Saad et al., 2016). In contrast, Kerley et al. (2017) conducted an RCT study including 38 children with ASD, which found that vitamin D3 did not affect RRBs after 5-month supplementation.

#### Folic Acid and Omega-3 Fatty Acid

The abnormal metabolism of folic acid is related to ASD (Castro et al., 2016), and folic acid deficiency have been found in the brains of individuals with ASD (Karin et al., 2017). Supplementing high doses of folic acid in maternal mice can significantly reduce RRBs of offspring (Di et al., 2021). A recent RCT study evaluated the effect of high-dose leucovorin supplementation in ASD. Forty-eight children with ASD and language barriers were randomly given leucovorin or placebo, and RRBs were significantly improved in the leucovorin group (Frye et al., 2018).

Omega-3 fatty acid is associated with mood disorders. Some preliminary studies suggested that Omega-3 fatty acid could effectively treat various mental disorders, such as ASD (Yehuda et al., 2005). Yui conducted a 12-week, 240 mg/day DHA + 240 mg/day arachidonic acid (ARA) intervention on 13 individuals with ASD, and significant improvements in RRBs were observed (Yui et al., 2012). However, the latest meta-analysis, including six studies, suggested no significant effect of omega-3 fatty acids (Zhou et al., 2021).

#### Other Supplements

Gastrointestinal problems and unique gut flora in individuals with ASD are related to the development and severity of ASD symptoms (Hughes et al., 2018). Changing gut flora is considered a promising treatment for related behavioral disorders. Seventeen children with ASD (3–16 years old) were supplemented with *Lactobacillus Plantarum* WCSF1. After a 12-week intervention, the behavioral score was significantly improved (Parracho et al., 2010). Additionally, some dietary patterns have shown advantages of RRBs in animal models (Castro et al., 2017; Lee et al., 2018a). Gluten-free diets (Ghalichi et al., 2016), casein-free diets (Lucarelli et al., 1995), and ketogenic diets (El-Rashidy et al., 2017) have been verified to have positive effects on ASD symptoms. However, another study reported no significant differences in RRBs (Harris and Card, 2012; Navarro et al., 2015). So, there are still uncertainties about the effects of dietary approaches. Further investigations are needed to confirm these dietary interventions’ specific efficacy and safety for RRBs with a larger sample.

### Other Treatment

Except for the above three intervention methods, many other emerging non-drug treatments targeting RRBs have been reported. Cell therapy indications have been expanded from hematological malignancies to other diseases. Cell therapy has shown preliminary safety and effectiveness in children with ASD. Moreover, transcranial magnetic stimulation (TMS) is used in various mental diseases with specific effects. Researchers began to explore its effects on ASD symptoms in recent years.

#### Cell Therapy

The studies of epigenetics, neuroimmunology, and neurobiology in ASD indicated that cell therapy was an effective approach for treating the core symptoms of ASD (Vaccarino et al., 2011; Siniscalco et al., 2012b; Liu et al., 2019; Zhang et al., 2021). Two outstanding features of stem cells are the intense immunosuppressive activity that allows them to be used in autologous or heterologous transplantation (Siniscalco et al., 2012a) and paracrine actions (Baraniak and McDevitt, 2010; Siniscalco, 2012). Stem cells usually synthesize and release a variety of cytokines, chemokines, and growth factors (Beyth et al., 2005; Siniscalco et al., 2012b), which can reduce the proinflammatory state observed in children with ASD (Gupta et al., 2010) and activate endogenous repair mechanism to recover the damaged function of related cells and tissues (Siniscalco et al., 2012b).

Stem cell therapy has shown benefits to RRBs in various ASD models. Intraventricular administration of mesenchymal stem cells (MSCs) significantly improved core ASD-like symptoms in BTBR mice, including social interaction and RRBs (Segal-Gavish et al., 2016). In addition, intranasal administration of human exosomes derived from mesenchymal stem cells (MSC-exos) was effective on all core ASD behaviors in two different mice (BTBR and *SHANK3* KO) (Perets et al., 2018, 2020). Transplantation of mesenchymal stem cells has been proven safe in many clinical trials (Gupta et al., 2010), and whether mesenchymal stem cells apply to individuals with ASD and clinically improve the ASD-like symptoms deserves to be further explored. Regarding clinical trials on cell therapy, Lv et al. conducted a single-center phase I/II trial to assess the safety and efficacy of combined transplantation of human cord blood mononuclear cells (CBMNCs) and umbilical cord-derived mesenchymal stem cells (UCMSCs) in 37 children with ASD (3–12 years of age). Individual transplantation of CBMNCs was demonstrated to remarkably decrease repetitive behaviors compared to the control group. In addition, combined transplantation of CBMNCs and UCMSCs showed better therapeutic effects (Lv et al., 2013). Similarly, Nguyen Thanh et al. (2021) found that transplantation of mononuclear cells of bone marrow combined with educational intervention showed that RRBs and hyperactivity were significantly reduced in children with ASD. These stem cell trials were proved excellent safety, with no safety issues noted during injection and the whole follow-up period (Lv et al., 2013; Nguyen Thanh et al., 2021). Significantly, the benefits of cell therapy to RRBs are necessary to be clarified because most clinical studies in ASD focused on social deficits rather than RRBs or regarded the alterations of RRBs as a secondary result (Bradstreet et al., 2014; Chez et al., 2018; Villarreal-Martínez et al., 2021).

There is still a long way before cell therapy becomes an approved treatment for RRBs in ASD. More in-depth and detailed studies on stem cell biology are necessary to understand the mechanism in RRBs. In addition, the exact dose, time, and site of stem cell infusion, as well as the fatal side effects and long-term safety, need to be further determined. For example, some researchers are concerned about intravenous administration because animal models have shown that it was difficult for transplanted cells to pass through organs (e.g., spleen and kidney) via intravenous administration (Steiner et al., 2012). Moreover, there is a correlation between the dose of transplanted stem cells and the subsequent clinical improvement (Rocha et al., 2002), which emphasized the importance of choosing the exact dose. What should not be ignored is ethical issues in stem cell studies. Such as the acquisition of stem cells, the safety of stem cell collection and administration, the tumorigenicity of stem cells, and other ethical risks similar to other clinical studies (Siniscalco, 2012). The life expectancy of children with ASD is close to normal, and potential risks of children’s medication are difficult to define. Therefore, interventional stem cell therapy is morally untenable unless more studies prove that the apparent benefits outweigh the risks (Yeo-Teh and Tang, 2021).

#### Transcranial Magnetic Stimulation

Transcranial Magnetic Stimulation is a non-invasive brain stimulation used to treat depression and other mental illnesses via changing the excitability of neural circuits and reorganizing the functions of cortex. Previous pilot studies reported positive effects of repeated TMS (rTMS) in individuals with ASD (Sokhadze et al., 2009; Casanova et al., 2012; Wang Y. et al., 2016). Irritability, hyperactivity, and RRBs were decreased in 27 participants with ASD after 18-rounds rTMS on the dorsolateral prefrontal cortex, and the latest study also drew a similar conclusion (Sokhadze et al., 2014; Abujadi et al., 2018). In addition, adults with autism and major depressive disorder reported improvements in repetitive behaviors after 25-session rTMS (Gwynette et al., 2020). A consensus statement of rTMS for ASD showed that rTMS was a potential treatment for ASD and suggested that existing studies have significant limitations, and more definitive studies needed to be conducted to clarify the safety and efficacy of rTMS in ASD (Cole et al., 2019).

## Perspective and Future Directions

Compared with studies targeting the social communication deficits in ASD, current evidence of RRBs is limited. As the core symptoms of ASD, the accurate assessment of RRBs is crucial. Individuals with ASD show remarkable differences in types and severity of RRBs, depending on different ages, genders, and functional statuses. Because of the high heterogeneity of RRBs, identifying additional subtypes of RRBs may be useful. In addition, researchers developed assessment tools based on male individuals with ASD and did not consider adequately specific RRBs of the female sample, so it is urgent to develop evaluation tools suitable for different clinical populations with excellent sensitivity and applicability. Salloum-Asfar et al. (2019) suggested that miRNA was a promising biomarker for ASD diagnosis and core symptom assessment. For example, the level of specific miRNA in saliva was positively correlated with the score of repetitive restricted behavior (Hicks et al., 2020). Whether miRNA can be used as a biomarker to assess the severity of RRBs needs further exploring.

Moreover, with the development of new technologies and methods, more specific mechanisms of RRBs will be discovered. RRBs are mostly considered as a secondary outcome in current studies. Thus, it is necessary to explore the relationship between neural circuitry and subtypes of RRBs via more specific assessment tools (e.g., RBS-R). Moreover, Van’t Westeinde et al. (2020) found that RRB-related structural alterations of striatal networks are more common in men, while abnormity of frontoparietal networks was more observed in females, which implied some differences in neural networks between male and female were omitted in the studies without enough female sample. Future studies of RRBs will include a larger female sample to reveal possible gender differences in neural circuitry related to RRBs. The related studies of the relationship between hyperdirect pathway and RRBs are scarce. The increasingly recognized importance of the hyperdirect pathway suggest it may play an essential role in RRBs. In addition, the connectivity between the basal ganglia and cerebellum is related to behavior control, but how aberrant connectivity affects RRBs is unclear in ASD. Therefore, there is a strong need to investigate structural and functional connectivity related to RRBs. To date, multiple RRBs-exhibiting ASD animal models have been developed. Focusing on common pathophysiological changes (e.g., abnormal immune function) in different animal models may provide some crucial insights. Furthermore, future studies on the potential neurobiology of reinforcement and habituation will also contribute to a better understanding of RRBs. It is worth noting that some conclusions are based on rodent models, and translation of these findings to RRBs in humans with ASD is difficult. So, the studies in primates may provide more evidence.

Lastly, how to intervene in the core symptoms of ASD is still the most important and meaningful issue for individuals with ASD. So far, behavioral intervention is still an essential part of treatment for RRBs. There are still three significant obstacles in psychopharmacology studies: (a) thus far, most trials have not found significant differences in primary endpoint suggesting insufficient effectiveness; (b) there are vast heterogeneities of clinical effects and side effects in different people; (c) the overlapping symptoms (e.g., anxiety and hyperactivity) and uncertain mechanism of RRBs make it challenging to find a drug aimed at specific targets. Even so, some medications, such as oxytocin and budesonide, seemed to show benefits to RRBs. The RCT studies with a larger sample are necessary to verify their efficacy and safety. Due to the limited number of studies, the specific efficacy and safety of supplementary therapy on RRBs remain unclear. As a promising treatment, cell therapy faces many scientific and ethical issues. In-depth and detailed studies of stem cell biology are required to help understand the mechanism of stem cells. The exact dose, time and site of stem cell infusion, the fatal side effects, and long-term safety should be determined in clinical trials.

## Author Contributions

JT drafted the manuscript. XG revised the manuscript. LY edited the language and the final version of the manuscript. All authors contributed to the article and approved the submitted version.

## Conflict of Interest

The authors declare that the research was conducted in the absence of any commercial or financial relationships that could be construed as a potential conflict of interest.

## Publisher’s Note

All claims expressed in this article are solely those of the authors and do not necessarily represent those of their affiliated organizations, or those of the publisher, the editors and the reviewers. Any product that may be evaluated in this article, or claim that may be made by its manufacturer, is not guaranteed or endorsed by the publisher.
